# A structured open dataset of government interventions in response to COVID-19

**DOI:** 10.1038/s41597-020-00609-9

**Published:** 2020-08-27

**Authors:** Amélie Desvars-Larrive, Elma Dervic, Nina Haug, Thomas Niederkrotenthaler, Jiaying Chen, Anna Di Natale, Jana Lasser, Diana S. Gliga, Alexandra Roux, Johannes Sorger, Abhijit Chakraborty, Alexandr Ten, Alija Dervic, Andrea Pacheco, Ania Jurczak, David Cserjan, Diana Lederhilger, Dominika Bulska, Dorontinë Berishaj, Erwin Flores Tames, Francisco S. Álvarez, Huda Takriti, Jan Korbel, Jenny Reddish, Joanna Grzymała-Moszczyńska, Johannes Stangl, Lamija Hadziavdic, Laura Stoeger, Leana Gooriah, Lukas Geyrhofer, Marcia R. Ferreira, Marta Bartoszek, Rainer Vierlinger, Samantha Holder, Simon Haberfellner, Verena Ahne, Viktoria Reisch, Vito D. P. Servedio, Xiao Chen, Xochilt María Pocasangre-Orellana, Zuzanna Garncarek, David Garcia, Stefan Thurner

**Affiliations:** 1 Unit of Veterinary Public Health and Epidemiology, University of Veterinary Medicine Vienna, Veterinaerplatz 1, 1210 Vienna, Austria; 2 Complexity Science Hub Vienna, Josefstaedter Strasse 39, 1080 Vienna, Austria; 3 Section for Science of Complex Systems, Medical University of Vienna, Spitalgasse 23, 1090 Vienna, Austria; 4 Unit Suicide Research & Mental Health Promotion, Center for Public Health, Department of Social and Preventive Medicine, Medical University of Vienna, Kinderspitalgasse 15, 1090 Vienna, Austria; 5 CERMES3, Ecole des Hautes Etudes en Sciences Sociales, 7 rue Guy Moquet, 94801 Villejuif, France; 6 Gender, Sexuality, Health, CESP, INSERM, Paris-Saclay University, Paris-Sud University, UVSQ, Hôpital Paul Brousse, 16 avenue Paul Vaillant Couturier, 94807 Villejuif, France; 7 Advanced Systems Analysis, International Institute for Applied Systems Analysis (IIASA), Schlossplatz 1, A-2361 Laxenburg, Austria; 8 Flowers project-team, National Research Institute for Digital Sciences (INRIA), 200 avenue de la Vieille Tour, 33405 Talence, France; 9 Institute of Electrodynamics, Microwave and Circuit Engineering, Vienna University of Technology, Gusshausstrasse 25, 1040 Vienna, Austria; 10 German Centre for Integrative Biodiversity Research (iDiv) Halle-Jena-Leipzig, Deutscher Platz 5E, 04103 Leipzig, Germany; 11 Institute of Psychology, Jagiellonian University, ul. Gołębia 24, 31-007 Kraków, Poland; 12 Institute for Social Studies, University of Warsaw, ul. Krakowskie Przedmieście 26/28, 00-924 Warsaw, Poland; 13 Fundación Naturaleza El Salvador, Research Department, Colonia Escalón, San Salvador, CP 1101 El Salvador; 14 Seshat: The Global History Databank, http://seshatdatabank.info/; 15 Santa Fe Institute, Santa Fe, NM 87501 USA

**Keywords:** Databases, Viral infection, Government

## Abstract

In response to the COVID-19 pandemic, governments have implemented a wide range of non-pharmaceutical interventions (NPIs). Monitoring and documenting government strategies during the COVID-19 crisis is crucial to understand the progression of the epidemic. Following a content analysis strategy of existing public information sources, we developed a specific hierarchical coding scheme for NPIs. We generated a comprehensive structured dataset of government interventions and their respective timelines of implementation. To improve transparency and motivate collaborative validation process, information sources are shared via an open library. We also provide codes that enable users to visualise the dataset. Standardization and structure of the dataset facilitate inter-country comparison and the assessment of the impacts of different NPI categories on the epidemic parameters, population health indicators, the economy, and human rights, among others. This dataset provides an in-depth insight of the government strategies and can be a valuable tool for developing relevant preparedness plans for pandemic. We intend to further develop and update this dataset until the end of December 2020.

## Abstract (summary)

**Table:** 

Measurement(s)	time at medical intervention • medical intervention
Technology Type(s)	digital curation • content analysis strategy of existing information sources
Factor Type(s)	non-pharmaceutical intervention • date
Sample Characteristic - Location	global

Machine-accessible metadata file describing the reported data: 10.6084/m9.figshare.12668792

## Background & Summary

Non-pharmaceutical interventions (NPIs), also known as public health and social measures (PHSM)^1^, aim to prevent the introduction of infectious diseases (preparedness and readiness measures), control their spread and reduce their burden on the health system (control measures). The general concept of containing the initial (exponential) spread of a disease is called “flattening the (epi-)curve”^2^. By reducing the growth rate of an epidemic, NPIs reduce the stress on the healthcare system and help gaining time to develop and produce vaccines and specific medications, which is of utmost importance in the case of emerging infectious diseases.

During the COVID-19 pandemic, governments have enforced a broad spectrum of interventions, under rapidly changing, unprecedented circumstances. Government responses to COVID-19 included the *laissez-faire* strategy, which implies doing little to nothing, the *herd immunity* strategy, which implies a few measures only or measures relying on voluntary compliance, and more *aggressive* approaches based on the implementation of a wide range of stringent NPIs, sometimes even limiting civil rights and liberty^3,4^. Government control policies have shown divergences in particular in the timeline of implementation and in the prioritization of the NPIs. In China for example, quarantine, social distancing, cordon sanitaire, and isolation of cases have been associated with improvements in the key epidemiological markers, including the number of infections and COVID-19-related deaths^5^. In Hong Kong and Taiwan, which experienced severe acute respiratory syndrome (SARS) epidemics in 2002–2003^6,7^, early government actions, strict social distancing measures, contact tracing, extensive and proactive testing, and high compliance of the population, have, to date, successfully mitigated the COVID-19 epidemic^8,9^. Following a *herd immunity* approach, similar to the one initially adopted by the UK government, the Swedish government did not introduce strict bans but formulated non-binding recommendations only (https://www.folkhalsomyndigheten.se/nyheter-och-press/). Predictive models, however, suggest that such a strategy might ultimately overwhelm the healthcare system^10^.

Poor control policies have potentially dramatic repercussions on public health. Although the need for data on country-based responses to COVID-19 was urgent and is still crucial, there is a limited opportunity to capture this information. Started in mid-March 2020, our project aims to generate a comprehensive structured dataset on government responses to COVID-19, including the respective time schedules of their implementation.

During the COVID-19 crisis, several data collection efforts related to NPIs have emerged (https://lukaslehner.github.io/covid19policytrackers/). Some of them focus on a specific type of interventions, e.g. the closure of educational institutions (https://en.unesco.org/covid19/educationresponse), travel restrictions (https://www.iata.org/en/programs/safety/health/diseases/), trade-related measures (https://www.wto.org/english/tratop_e/covid19_e/trade_related_goods_measure_e.htm), or measures to ensure continuity of supply of personal protective equipment and critical medical products (http://www.wcoomd.org/en/topics/facilitation/activities-and-programmes/natural-disaster/list-of-countries-coronavirus.aspx), whereas others encompass a larger range of NPIs. In this paper, we also show how we distinguish our work from other concomitant initiatives.

In the context of the current COVID-19 health crisis, open knowledge^11^ and data sharing are crucial to understand and help to mitigate the pandemic. In this article, we document and share the methodologies, tools and approaches used to produce the *Complexity Science Hub COVID-19 Control Strategies List* (CCCSL) dataset following the principles of open science. We provide a detailed description of the dataset and present examples of how it can provide insights into the global government response to COVID-19.

The dataset is readily usable for modelling and machine learning analyses and exhibits a great analytical flexibility^12^. In particular, researchers have leveraged on the hierarchical structure and the granularity of the data to disentangle the individual impacts of the NPIs on the reduction of the effective reproduction number through a top-down approach (from theme to code). Results show that social distancing measures, travel restrictions, but also active risk communication, play a major role in containing the epidemic. The study further distinguishes the impact of different levels of implementation of some specific measures, e.g. those related to face covering^12^.

Considering the imperative necessity for data on government interventions, we released version 1 of the dataset on 2 April 2020. Version 2, displaying a consolidated coding scheme, is available since 7 May 2020. We also provide user-friendly documentation and materials (codes, visualisation interface, and library of sources) along with the dataset, which allow a maximum understanding of the data and promote its use among non-experts. The dataset is not complete and we continuously update it with new available records. Depending on resources, updates are planned until the end of December 2020.

## Methods

We used a content analysis^13–15^ strategy of existing information sources to develop a hierarchical coding scheme specific to NPIs implemented to mitigate the burden of COVID-19. First, based on a literature review on community mitigation strategies and expert knowledge, eight themes (thereafter called level 1 (L1) in the coding scheme) were identified and labelled: (i) Case identification, contact tracing and related measures, (ii) Environmental measures, (iii) Healthcare and public health capacity, (iv) Resource allocation, (v) Risk communication, (vi) Social distancing, (vii) Travel restriction, and (viii) Returning to normal life. A definition for each theme is provided in the Online-only Table 1. At the start of our project, there were no previously published studies on NPIs against COVID-19 to be used as a reference for developing the labelling and coding scheme. Therefore, a list of NPIs that have been already implemented by different governments at this time (mid-March 2020) was compiled, that served as a preliminary template to generate *a priori* categories within a hierarchical coding scheme. Strategies that could provide assistance to the population (e.g., related to financial support or food supply) or that may encourage compliance with the measures (e.g. resource allocations, risk communication) were also included. Listed interventions were then assigned to one of the eight themes defined above. The specific details and descriptions of each NPI were coded into *a priori* categories (thereafter called level 2 (L2) in the coding scheme), and into subsequent *a priori* subcategories and codes whenever needed (thereafter called level 3 (L3) and level 4 (L4) in the coding scheme, respectively). Discrepancies in code assignments were discussed within the coding team and were resolved by consensus. The objective of this hierarchical coding scheme for NPIs was to standardize the data collection and obtain a structured dataset that uses a consistent taxonomy, and therefore, promotes common understanding.

On 19 March 2020, we set up a platform for students, researchers, and volunteers to collect data on the NPIs implemented by the governments for preventing and limiting the spread of COVID-19, including the time schedules for the implementation. Data collectors received clear instructions on the objective of the project and indications on how to proceed for data collection. Data collectors were asked to use the template of *a priori* themes, categories, subcategories, and codes or to refer to the data curators if a measure could not be coded using this *a priori* coding system. Therefore, throughout the data collection process, new categories, subcategories, and codes emerged, derived directly from the text data sources. The emergent (inductive) categories and subcategories were openly coded by the data collectors or by the data curators. In a second step, inductive categories and subcategories were compared together and in relation to the entire dataset to detect co-occurrences (codes that partially or completely overlap) and redundancies. Codes with the same meaning were aggregated^16^. The categories and subcategories were tightened up to the point that maximized mutual exclusivity and exhaustiveness^15^. This resulted in a Master List of Codes (a list of all the codes that were developed and used in the study), including the curated *a priori* and inductive coding categories. The Master List replaced the *a priori* template for categorisation of the measures during data collection. It was shared with the data collectors via a Google spreadsheet and updated daily.

Different public sources were used to populate, update and curate the dataset, including official government sources, peer-reviewed and non-peer-reviewed scientific papers, webpages of public health institutions (World Health Organization, Centers for Disease Control and Prevention, and European Centre for Disease Prevention and Control), press releases, newspaper articles, and government communication through social media. We collected data on the following: (i) country, (ii) state/region (when measures were implemented at subnational-level), (iii) date of implementation of the measure, (iv) implemented measure coded following the four-level classification scheme described above (theme, category, subcategory and code), and (v) source. For each country, data were preferentially collected in the language of the country by native data collectors (i.e. Austria, Belgium, Bosnia and Herzegovina, Brazil, Canada, Croatia, Czech Republic, Ecuador, El Salvador, France, Germany, Ghana, Honduras, Hong Kong, India, Italy, Kazakhstan, Kosovo, Kuwait, Mauritius, Mexico, Montenegro, North Macedonia, New Zealand, Poland, Portugal, Ireland, Romania, Senegal, Serbia, Spain, Syria, Taiwan, and United Kingdom). If this was not possible, Google Translate was used to translate documents^17^. All records were hand-coded.

## Data Records

A static copy of the dataset has been archived in figshare^18^, including all NPIs recorded as of time of submission (17 July 2020), spanning the period 31 December 2019 to 15 July 2020. A dynamic version of the dataset, which is planned to be continually updated, can be accessed via GitHub: https://github.com/amel-github/covid19-interventionmeasures or from Google Drive: https://drive.google.com/open?id=1041U8iWPDSGI6KHIn9Dg7THkXIo3-gui, in CSV format. Each of the rows represents a single individual NPI and is identified by a unique ID. The Master List of Codes is also available (an additional Master List file displays the hierarchical relationship between each pair of parent/child codes, i.e. L1-L2, L2-L3, and L3-L4, and the number of times each pair occurs in the dataset). We also provide a Glossary of Codes, which gives the definition of each theme, category, subcategory, and code. An online interactive tool, which enables to visualise the dataset hierarchical structure and codes, completes the description of the dataset. It is accessible at: http://covid19-interventions.com/CCCSLgraph/. We have also established a GitHub repository available at: https://github.com/amel-github/CCCSL-Codes and provide codes^19^ for importing, exploring and visualising the data into R^20^. Furthermore, for purposes of transparency of data collection and to motivate collaborative validation process as well as a large use and development of the dataset, an open library is available, that contains all sources used to collect the data: https://www.zotero.org/groups/2488884/cccsl_covid_measure_project (>3,100 data sources are included as of date of submission). In order to leverage on the potential of crowdsourcing for populating and curating the CCCSL dataset, we have launched a webpage dedicated to this project at: http://covid19-interventions.com/ where contributors can fill up a Google Form at: https://bit.ly/2KsYOTn, if they wish to correct entries, add a measure, and/or provide a feedback.

The dataset contains the following fields:

**ID –** Unique identifier for each individually implemented measure. ID is also used in the Google Form to report erroneous entries.

**Country –** The country where the measure was implemented.

**ISO3 –** Three-letter country code as published by the International Organization for Standardization.

**State** – Subnational geographic area. State where the measure was implemented; the country name otherwise. Used for Germany, India, and USA.

**Region –** Subnational geographic area (e.g. region, department, municipality, city) where the NPI has been locally implemented (i.e. the measure was not implemented nationwide as of the mentioned date). The country or the state name otherwise (i.e. measure implemented nationwide).

**Date –** Date of implementation of the NPI. Date of announcement was used when the date of implementation of the NPI could not be found and this was specified in the field *Comment*.

**L1_Measure –** Theme (L1 of the classification scheme). Eight themes were defined (see Online-only Table 1).

**L2_Measure –** Category (L2 of the classification scheme). Online-only Table 1 provides the list of the categories for each theme.

**L3_Measure –** Subcategory (L3 of the classification scheme). Provides detailed information on the corresponding category (L2).

**L4_Measure –** Code (L4 of the classification scheme). Corresponds to the finest level of description of the measure.

**Status** – Indicates whether the measure is a prolongation of a previously implemented measure (“Extended”) or not (“”).

**Comment –** Provides the description of the measure as found in the text data source, translated into English. This field allows to judge the quality of the label for the different levels of the coding scheme and enables to re-assign the measure to the correct theme/category/subcategory/code in case of error or misinterpretation by the data collector^21^. When available, duration of the restriction, as officially announced, is mentioned in this field.

**Source –** Provides the reference for each entry, i.e. URL. Enables to trace back potential changes in the meaning of the label during the translation^21^. Enables to access the description of the measure in the source language and/or to access to the information as it was dispatched originally.

**Fig. 1:**
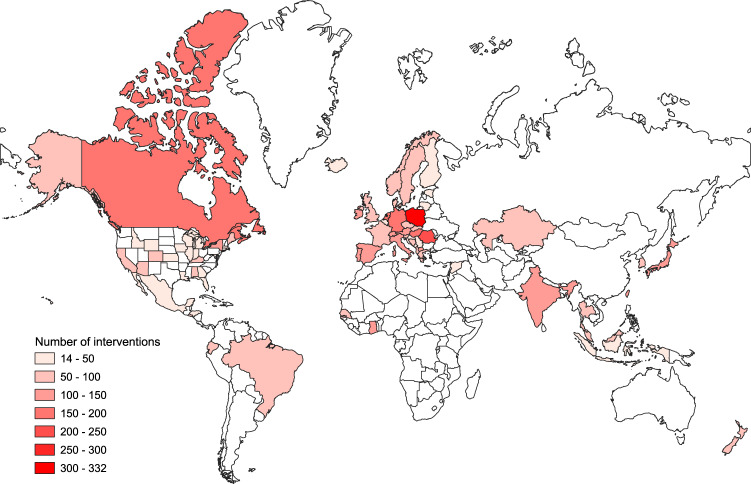
Geographical coverage of the CCCSL and total number of recorded NPIs that were implemented in each country to control the spread of COVID-19. As of date of submission, the dataset includes 56 countries and dates of NPI implementation range from 31/12/2019 to 15/07/2020.

As of date of submission, the CCCSL dataset included information for 6,068 government interventions, from 56 countries, including 33 European countries, 12 Asian countries, five South American countries, two North American countries, one Oceanian country, three African countries, and the Diamond Princess cruise ship. Regarding the USA, data are available at the state level for 24 states. Figure 1, Table 1, and Online-only Table 2 summarize the dataset. A description of the measures grouped by theme (L1) for each country can be computed from the published codes^19^ (https://github.com/amel-github/CCCSL-Codes).

**Table 1:** Summary of the government interventions recorded in the CCCSL at level 1 (themes) of the coding scheme.

Theme (L1)	Number of records	Frequency
Case identification, contact tracing and related measures	540	0.09
Environmental measures	62	0.01
Healthcare and public health capacity	808	0.13
Resource allocation	958	0.16
Returning to normal life	316	0.05
Risk communication	1,074	0.18
Social distancing	1,673	0.28
Travel restriction	615	0.10

As of date of submission, the dataset includes 56 countries and dates of NPI implementation range from 31/12/2019 to 15/07/2020.

## Technical Validation

After the initial data entry, the dataset was checked manually by the data curators. For each measure, concordance between L1, L2, L3, and L4 was checked. Moreover, the unique combinations of L1, L2, L3, and L4 were extracted and controlled for consistency. Typographical and coding errors were minimized through a manual process. We initiated a collaborative curation platform relying on internal and external collaborators who exchanged through Slack, GitHub, Skype, and via emails. This extended effort enabled us to correct typographical and coding errors, to remove line breaks, and to homogenize the dataset for universal use in different programming languages.

Beyond manual validations, we performed a technical validation step to detect possible duplicates. Using the *dplyr* package for the R Programming Language^22^, we identified any duplicate entries in the vector composed of country, region, date, and the codes from L1 to L4. Those entries were flagged as possible duplicates and reviewed by hand by two curators, ensuring that the dataset does not contain duplicated entries. An R script to reproduce this step is provided at: https://github.com/amel-github/CCCSL-Codes.

While an important effort has been made for standardizing the records, the four-level-*a priori* coding scheme originally proposed showed limitations. First, the existing classifications of NPIs are discordant^23–25^. We proposed an original classification scheme that best fitted our (emergency) needs and the specificity of the COVID-19 pandemic, but this scheme may be subjected to revisions in the future. Secondly, some NPIs have been uniquely implemented (e.g. the deportation of Chinese workers by the Kazakh government), which complicated the coding and categorisation process.

Access to the information from government or other official sources may be compromised if not performed timely. Indeed, several governments or national health agencies regularly update their webpage to provide the latest information to the public. Therefore, if sources are not consulted timely, previous content (i.e. previous restrictions and measures) might not be visible straight away and data will have to be retrieved indirectly or from archived websites, which eventually slows down the data collection process and may lead to missing data. Furthermore, while native speakers were recruited whenever possible for data collection, transliteration or translation errors may have occurred when extracting data from Google Translate translations.

Lastly, when using the data for epidemiologic or economic modelling, the absence of an “End date” data element might be a limitation. However, this data cannot be captured for each kind of NPI, e.g. “Increase of healthcare workforce” or “Work safety protocol”. We propose an alternative approach that leverages on the theme “Returning to normal life” and record individually all (i) variations in, (ii) conditions of, and (iii) adaptive measures to the gradual lifting of the restrictions (e.g. re-opening of shops > 400 m², re-opening of classes with examination, weddings allowed if the number of attendees is < 100, etc.). By providing data on each step of the phase-out process, the coding scheme allows therefore to retrieve even more specifically (but indirectly) the “End date” for each NPI (to the best of our knowledge, only the CoronaNet dataset provides a “End date” data element, although as of date of writing, for 30% of the interventions only^26^).

We plan to maintain the quality level of the dataset with regular updates on the countries currently described. Furthermore, we plan to increase the geographic coverage of the dataset, prioritizing large countries (e.g. China, US states not already covered, and Australia), those with a high number of reported cases (e.g. Vietnam, Iran, Turkey, Russia, Israel, Peru, Chile, Pakistan, Philippines, Saudi Arabia), and those where the epidemic is rising and which may suffer from a data gap (i.e. African and South American countries). The same technical procedures and the classification scheme described above will be applied to any new information to be included in the dataset. Future versions will be subjected to extensive data validation processes. We plan to stabilise the hierarchical coding scheme for NPIs implemented to contain COVID-19 within six months, including measures related to the lifting of the restrictions and adaptive measures that accompany them.

## Usage Notes

The aim of this work is not only to improve the current knowledge on country-based interventions implemented to mitigate the burden of COVID-19, but also to characterise the political, public health, and economic strategies of the governments worldwide. Combined with publicly available data on the number of confirmed cases, recovered cases, and deaths, the CCCSL dataset makes it possible to assess the effectiveness of the control policies on the COVID-19 epidemic, e.g. the epidemic growth rate or the daily reproduction numbers^12^. The standardized coding facilitates an inter-country comparison of government responses. The dataset can further benefit the risk assessment of lifting some restrictions and the development of exit strategies. It can also become an essential data source in the aftermath of the first wave of COVID-19, to guide government control policies anticipating a potential second wave of cases. We envision the CCCSL dataset to become a timely valuable and long-lasting data source for assessing the impact of the NPIs on global public health indicators, the economy, and human rights, among others. We provide below two examples of data usages that give an insight into the responsiveness and aggressiveness of the governments in their management of the COVID-19 crisis.

### Mapping the timeline of government interventions during the epidemic

**Fig. 2:**
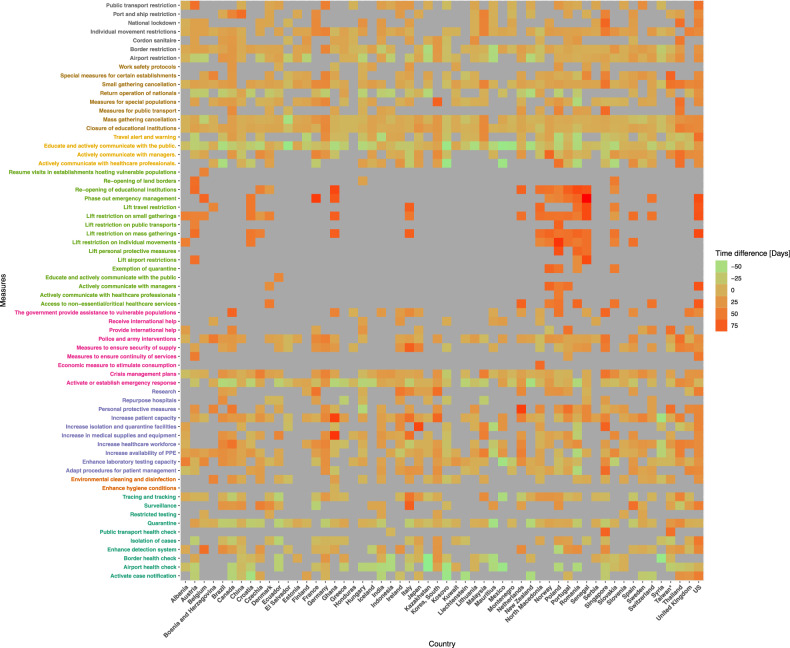
Heat map of the dates of implementation of the NPIs recorded in the CCCSL at the level 2 of the hierarchical coding scheme (categories) in 56 countries. Time is in epidemic age with t_0_ = day when 10 cases were reported (http://covid19-interventions.com/CountryMeasuresHeatmap.svg).

We propose to visualise the time-series of the dates of implementation of the NPIs recorded in the CCCSL at the level 2 of the hierarchical coding scheme (categories) in the 56 countries using a heat map (Fig. 2). In order to highlight country-based differences in the timeline of implementation, we used the epidemic age instead of calendar time. For a given day, t, in a certain country, the epidemic age is defined as the time difference, t-t_0_, measured in days, where t_0_ is the first day when the number of confirmed cases was greater or equal to 10. The time-series data of the number of COVID-19 cases was retrieved from the COVID-19 Data Repository by the Johns Hopkins University Center for Systems Science and Engineering (JHU CSSE) at: https://github.com/CSSEGISandData/COVID-19.

### Country-cluster analysis of the government control strategies

**Fig. 3:**
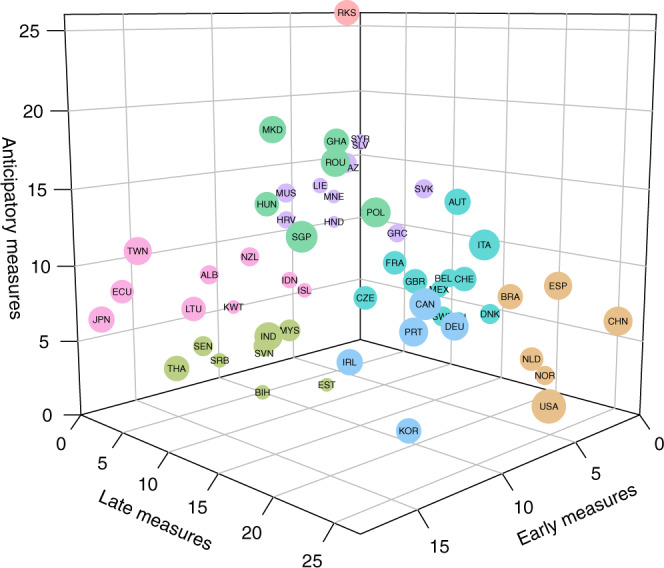
Country-cluster analysis based on the number of mandatory government interventions and respective dates of implementation, as calculated using the epidemic age (t_0_ = day when 10 cases were reported) (http://covid19-interventions.com/CountryClusters.html).

In order to partition the countries based on the aggressiveness (number of NPIs) and responsiveness (timeline) of their control strategy, we applied a k-means clustering. We focused on mandatory government interventions (i.e. the theme “Risk communication” was not included) recorded in the CCCSL at the level 2 of the hierarchical coding scheme (categories) that appeared in at least 15 countries, leading to a total number of 40 categories. The clustering algorithm uses the date of implementation of the measures in each country to build a feature vector based on the epidemic age (see above). We considered “anticipatory measures” as those implemented before day when 10 cases were reported; “early measures” as those implemented at the beginning of the epidemic, i.e. between the day when 10 cases were reported and the day when 200 cases were reported; and “late measures” as those implemented at a later stage of the epidemic, i.e. after the day when 200 cases were reported. The algorithm takes also into account the number of measures implemented at these different stages of the epidemic. The time-series data of the number of COVID-19 cases was retrieved from the COVID-19 Data Repository by the JHU CSSE (https://github.com/CSSEGISandData/COVID-19). The optimal number of clusters, k, was determined using the elbow method^27^. Briefly, this method consists in running k-means clustering on the dataset for a range of values of k (set here from 1 to 15), and for each value of k calculates the sum of squared errors (SSE). We then plotted the SSE for each value of k and identified the best value of k where the line chart looks like an arm (“elbow”). As of date of publication (static version 2020-07-12, 56 countries) the best value of k was eight, explaining 82.8% of the variance (Fig. 3). An interactive version of Fig. 3 is available online at: http://covid19-interventions.com/CountryClusters.html.

### Contextualising the project

During the COVID-19 crisis, other projects have concomitantly tracked data on government policies (interchangeably named NPIs^18^ or government(s’) responses^26,28,29^ or government measures^30^ or PHSM^31^ or policy actions^26^). We report here on five of them^26,28–31^ in order to contextualise our work. The comparison indicates similarities and differences among the NPI trackers and highlights how the CCCSL contributes to the global effort against COVID-19. Supplementary Information 1 outlines the main characteristics of the six datasets (including the CCCSL^18^).

The core value-added of the CCCSL dataset is the remarkable granularity of the data on NPIs (e.g. seven categories of travel restriction are reported, further divided into more than 50 subcategories) and the use of self-explanatory codes, which, completed with the Glossary of Codes, makes the dataset readily intelligible. As of date of submission, the dataset displays eight themes, 63 categories, >500 subcategories, and >2,000 codes.

With regard to the geographic unit, two datasets record data at the country level^28,29^ whereas four record data at a finer administrative scale^18,26,30,31^. One dataset uses a binary code (1/0) to assess the presence/absence of the NPIs^28^, another one uses an Likert-like scale to further differentiate the level of implementation^29^, whereas the others use a coding system based on words or short phrases that assign a summative attribute to the data^18,26,30,31^. Moreover, the aggregation scheme and, sometimes, the semantic of the NPIs diverge widely between the datasets. For example, the CoronaNet dataset^26^ groups school closure together with lockdown measures whereas the CCCSL^18^ and the ACAPS^30^ datasets classify school closure in the theme “Social distancing”. Regarding the restriction on individual movement, this measure is labelled *“Partial lockdown”* in the ACAPS dataset^30^, *“Household confinement”* in the HIT-COVID dataset^31^, *“Lockdown applies to all people”* in the CoronaNet dataset^26^, and *“Movements for non-essential activities forbidden”* in the CCCSL dataset^18^. Overall, these projects are independent of each other and the specific research question should indicate which one(s) to use. Harmonizing and integrating the different datasets could help accelerate epidemiological understanding on COVID-19 and the development of relevant preparedness plans for pandemic. The World Health Organization is currently making an important effort in this regard^1^.

## Supplementary information

Supplementary Information 1

## Data Availability

A live version of this project is accessible on GitHub at: https://github.com/amel-github/covid19-interventionmeasures. The codes used to describe the CCCSL dataset and the codes used to explore the CCCSL dataset are written in R language^19^. They are available at: https://github.com/amel-github/CCCSL-Codes. Please refer to the README file in the code release for further instructions.

## Online-only Tables

**Online-only Table 1:** Definition of the eight themes (L1) used to classify the NPIs and associated categories (L2).

Theme	Definition	Associated categories
Case identification, contact tracing and related measures	Measures aiming at identifying cases (testing, surveillance), tracing the contacts (i.e. identification and follow-up of persons who may have come into contact with an infected person^25^), implementing isolation for cases and quarantine for contacts and suspected cases (including tracking).	[1] Activate case notification [2] Airport health check [3] Border health check [4] Enhance detection system [5] Isolation of cases [6] Public transport health check [7] Quarantine [8] Restricted testing [9] Surveillance [10] Tracing and tracking
Environmental measures	Measures aiming at reducing the transmission of COVID-19 through shared environment and surfaces. Those measures include the routine cleaning of frequently used surfaces, clothes and objects; minimizing the sharing of objects; and ensuring appropriate ventilation^32^.	[1] Enhance hygiene conditions [2] Environmental cleaning and disinfection
Healthcare and public health capacity	Public health capacity under the International Health Regulations (IHR) (2005) is defined as the indispensable, fundamental actions that are the primary responsibility of each State Party for achieving the goal of national health security, i.e. to prevent the spread of diseases and to detect and investigate health risks in the community by efficient multisectoral action (e.g. laboratory services and national, regional and global networks^33^. Healthcare capacity refers to patient capacity, healthcare workforce capacity, laboratory capacity, availability of medical supplies and equipment.	[1] Adapt procedures for patient management [2] Enhance laboratory testing capacity [3] Increase availability of PPE [4] Increase healthcare workforce [5] Increase in medical supplies and equipment [6] Increase isolation and quarantine facilities [7] Increase patient capacity [8] Personal protective measures [9] Repurpose hospitals [10] Research
Resource allocation	Resource allocation covers the whole range of operations involved in the allocation of budgets, deployment of resources, and distribution of goods for controlling a disease outbreak (these measures are most often carried out by national, regional, or even local authorities). This theme also includes the use of military and/or police forces to ensure compliance with the implemented NPIs. For example, under the IHR (2005), each State Party is required to develop, strengthen and maintain core public health capacities for surveillance and response by using existing national resources, such as the national plans for pandemic preparedness^33,34^.	[1] Activate or establish emergency response [2] Crisis management plans [3] Economic measure to stimulate consumption [4] Measures to ensure continuity of services [5] Measures to ensure security of supply [6] Police and army interventions [7] Provide international help [8] Receive international help [9] The government provide assistance to vulnerable populations
Returning to normal life	Measures supporting the return to normal life, i.e. lifting of restrictive measures (e.g. lift of quarantine, re-opening of schools, re-opening of shops) and adaptive measures.	[1] Access to non-essential/critical healthcare services [2] Actively communicate with healthcare professionals [3] Actively communicate with managers [4] Educate and actively communicate with the public [5] Exemption of quarantine [6] Lift airport restrictions [7] Lift personal protective measures [8] Lift restriction on individual movements [9] Lift restriction on mass gatherings [10] Lift restriction on public transports [11] Lift restriction on small gatherings [12] Lift travel restriction [13] Phase out emergency management [14] Re-opening of educational institutions [15] Re-opening of land borders [16] Resume visits in establishments hosting vulnerable populations
Risk communication	All implemented methods for using communication strategically to achieve positive behavioural and social results. Risk communication includes health education, health literacy, health promotion, risk communication and social mobilization^25^. We have included in this category all voluntary, non-compulsory, recommended measures of any kind.	[1] Actively communicate with healthcare professionals [2] Actively communicate with managers [3] Educate and actively communicate with the public [4] Travel alert and warning
Social distancing	Social distancing refers to methods for reducing frequency and closeness of contact between people in order to decrease the risk of transmission of diseases. Examples of social distancing include cancellation of public events such as concerts, sports events, or movies, closure of office buildings, schools, and other public places, and restriction of access to public places such as shopping malls or other places where people gather^34^.	[1] Closure of educational institutions [2] Mass gathering cancellation [3] Measures for public transport [4] Measures for special populations [5] Return operation of nationals [6] Small gathering cancellation [7] Special measures for certain establishments [8] Work safety protocols
Travel restriction	Travel restriction encompasses all measures that aim to limit the (free) movements of individuals, e.g. restrictions on the entry and exit of a country, internal travel restrictions, border closure, cordon sanitaire, and national lockdown.	[1] Airport restriction [2] Border restriction [3] Cordon sanitaire [4] Individual movement restrictions [5] National lockdown [6] Port and ship restriction [7] Public transport restriction

PPE: Personal Protective Equipment.

**Online-only Table 2:** Top 20 most frequent categories (L2) of NPIs implemented to control the spread of COVID-19. As of date of submission, the dataset includes 56 countries and dates of NPI implementation range from 31/12/2019 to 15/07/2020.

Rank	Category (L2)	Number of records	Frequency	Examples of associated subcategories (L3)
1	Educate and actively communicate with the public	788	0.13	Answer to questions; Call for return of nationals living abroad; Direct advice to vulnerable populations; Discourage non-essential travels; Encourage donation for covid19 crisis; Encourage hand hygiene; Encourage self-initiated quarantine; Encourage vaccination against respiratory diseases other than covid19; Information campaign; Promote hygiene measures and social distancing; Promote self-initiated isolation of people with mild respiratory symptoms; Respiratory etiquette.
2	Mass gathering cancellation	631	0.10	Closure of discotheques; Closure of markets; Closure of non-essential public places; Conferences, meetings, trade fairs, etc.; Cultural places and events; Implement e-learning; Indoor activities; Measures for elections; Outdoor activities; Places of worship; Sport events; Requirement on the number of persons allowed.
3	Crisis management plans	442	0.07	Administrative procedures facilitated; Develop all-of-society and business continuity plans; Financial aid for health system; Funding for non-profit institutions; Measures for special populations; Repurpose government plans; State aid, taxation and social security.
4	Closure of educational institutions	332	0.05	Restrictions on exams; Closure of kindergartens; Closure of primary and secondary schools; Closure of universities; Precise complete or partial closure.
5	Small gathering cancellation	312	0.05	Closure of non-essential shops; Closure of restaurants/bars/ cafes; Closure of short-term accommodation, Restriction on private and familial events; Complete prohibition of gathering; Mandatory home office; Requirement on the number of persons allowed.
6	Activate or establish emergency response	214	0.04	Declare state of emergency; Exceptional change to work law; Relaxation of data protection law; Risk management plan.
7	Border restriction	195	0.03	Border control; Entry ban to non-citizens; Entry ban to people from China; Entry ban to people from high-risk areas other than China; Entry ban to refugees; Force departure of Chinese nationals; Land border controls; Total entry ban; Travel ban to high-risk areas.
8	Quarantine	180	0.03	Contact persons; Incoming residents; Incoming travellers; Incomings from high-risk areas; Nationals coming from high-risk areas; Suspected cases.
9	Increase availability of PPE	153	0.03	Face masks; Hand sanitizers; Increase domestic production of PPE; PPE for healthcare professionals; PPE other than face masks; Prohibition of export of protective personal equipment.
10	Measures for special populations	153	0.03	Measures to limit contact to hospital patients; Measures to limit contact to long-term care facilities; Measures to limit contact to the elderly; Measures to protect vulnerable populations; More exposed professionals (not healthcare).
11	Airport restriction	148	0.02	Airports closed; Cancellation of domestic flights; Cancellation of international flights; Landing bans on aircrafts from high risk areas; Some airports dedicated to receive international flights.
12	Increase healthcare workforce	147	0.02	Exception to work law allowed; Incentives for healthcare workers; Mobilization of domestic resources for health; Movement restriction of healthcare professionals; Train medical staff especially for covid-19.
13	Actively communicate with managers	143	0.02	Call on the religious authorities; Encourage event cancellation; Flexibilization of working hours/school hours; Guidelines; Recommendations for work safety protocols; Risk assessment before an event.
14	Special measures for certain establishments	132	0.02	Decongestion of administrative institutions; Entertainment venues; Essential businesses and operations; Implement the 2 m distance; Non-essential shops; Places of worship; Prisons and youth detention centers; Restaurants; Sport centers; Veterinary clinic.
15	Individual movement restrictions	129	0.02	Curfew; Movements for non-essential activities forbidden; Non-essential travels abroad/out-of-state forbidden; Partial restriction on movements; Prohibition of moving out the municipality of residence; Restrictions on the movements of children; Segmentation of the population.
16	Police and army interventions	121	0.02	Against dissemination of fake news; For cybersecurity; Increase police/military forces; To collect data and samples for tests; To support essential workers; To support law enforcement/sanction in case of no compliance; To support the population.
17	Travel alert and warning	114	0.02	Travel alert level 1 to 6 (concerned country is specified); Warning against travel to and return from high-risk areas.
18	Increase patient capacity	105	0.02	Beds; Emergency hospitals; Increase ICU capacity; Increase medical consultation capacity; Medicalise nursing home; Postponement of non-essential care and non-urgent operations in hospitals
19	Measures to ensure security of supply	97	0.02	Ban export of necessity goods; Fixing price for medical supplies; Fixing price for necessity goods; Fixing price for specific protective products; Increase delivery capacity for medical supplies; Increase delivery capacity for necessity goods; Insure access to gas/electricity/water/telecommunications; Traffic enactments.
20	Airport health check	90	0.01	Health certificate requested at airport; Health questionnaire in the plane or at the airport; Health screening at the airport; Specific health channel for travellers; Temperature screening at airport; Test travellers with fever or symptoms.

## References

[1] World Health Organization. Tracking Public Health and Social Measures A Global Dataset. https://www.who.int/emergencies/diseases/novel-coronavirus-2019/phsm (2020).

[2] Anderson RM, Heesterbeek H, Klinkenberg D, Hollingsworth TD (2020). How will country-based mitigation measures influence the course of the COVID-19 epidemic?. Lancet.

[3] Ugarov, A. Inclusive costs of NPI measures for COVID-19 pandemic: three approaches. Preprint at 10.1101/2020.03.26.20044552 (2020).

[4] Studdert DM, Hall MA (2020). Disease control, civil liberties, and mass testing — Calibrating restrictions during the Covid-19 pandemic. N. Engl. J. Med..

[5] Pan A (2020). Association of public health interventions with the epidemiology of the COVID-19 outbreak in Wuhan, China. JAMA.

[6] Chen K-T (2005). SARS in Taiwan: an overview and lessons learned. Int. J. Infect. Dis..

[7] Hung LS (2003). The SARS epidemic in Hong Kong: what lessons have we learned?. J. R. Soc. Med..

[8] Cowling BJ (2020). Impact assessment of non-pharmaceutical interventions against coronavirus disease 2019 and influenza in Hong Kong: an observational study. Lancet Public Health.

[9] Wang CJ, Ng CY, Brook RH (2020). Response to COVID-19 in Taiwan: Big data analytics, new technology, and proactive testing. JAMA.

[10] Rocklov, J. COVID-19 health care demand and mortality in Sweden in response to non-pharmaceutical (NPIs) mitigation and suppression scenarios. Preprint at 10.1101/2020.03.20.20039594 (2020).

[11] Molloy JC (2011). The open knowledge foundation: open data means better science. PLoS Biol..

[12] Haug, N. *et al*. Ranking the effectiveness of worldwide COVID-19 government interventions. Preprint at 10.1101/2020.07.06.20147199 (2020). 10.1038/s41562-020-01009-0 33199859

[13] Vaismoradi M, Turunen H, Bondas T (2013). Content analysis and thematic analysis: implications for conducting a qualitative descriptive study. Nurs. Health Sci..

[14] Erlingsson C, Brysiewicz P (2017). A hands-on guide to doing content analysis. Afr. J. Emerg. Med..

[15] Weber, R. *Basic Content Analysis* 2 edn (SAGE Publications, Inc, 1990).

[16] Gläser, J. & Laudel, G. Life with and without coding: Two methods for early-stage data analysis in qualitative research aiming at causal explanations. *Forum Qual. Soc. Res*. **14**, Art. 5 (2013).

[17] Windsor LC, Cupit JG, Windsor AJ (2019). Automated content analysis across six languages. PLoS One.

[18] Desvars-Larrive A (2020). figshare.

[19] Desvars-Larrive, A., Dervic, E., Haug, N. & Garcia, D. A structured open dataset of government interventions in response to COVID-19–Codes for exploration and visualisation. *Zenodo*, 10.5281/zenodo.3949808 (2020). 10.1038/s41597-020-00609-9 PMC7452888 32855430

[20] R Core Team. R: A language and environment for statistical computing, https://www.R-project.org/ (R Foundation for Statistical Computing, Vienna, Austria, 2020).

[21] Vaismoradi M, Jones J, Turunen H, Snelgrove S (2016). Theme development in qualitative content analysis and thematic analysis. J. Nurs. Educ. Pract..

[22] Wickham, H., François, R., Henry, L. & Müller, K. *dplyr:* a grammar of data manipulation, https://CRAN.R-project.org/package=dplyr (2020).

[23] Centers for Disease Control and Prevention. *Interim Pre-Pandemic Planning Guidance: Community Strategy for Pandemic Influenza Mitigation in the United States: Early, Targeted, Layered Use of Nonpharmaceutical Interventions* (Stephen B. Thacker CDC Library, 2007).

[24] European Centre for Disease Prevention and Control. *Technical Report. Guide to Revision of National Pandemic Influenza Preparedness Plans - Lessons Learned From the 2009 A(H1N1) Pandemic* (ECDC, Stockholm, 2017).

[25] World Health Organization. *Non-Pharmaceutical Public Health Measures for Mitigating the Risk and Impact of Epidemic and Pandemic Influenza* (World Health Organization, 2019). 42118904

[26] Cheng C, Barceló J, Hartnett A, Kubinec R, Messerschmidt L (2020). COVID-19 Government Response Event Dataset (CoronaNet v1.0). Nat. Hum. Behav..

[27] Yuan C, Yang H (2019). Research on K-value selection method of K-means clustering algorithm. J..

[28] Porcher S (2020). OPENICPSR.

[29] Hale, T., Webster, S., Petherick, A., Phillips, T. & Kira, B. Oxford COVID-19 Government Response Tracker, Blavatnik School of Government. https://www.bsg.ox.ac.uk/research/research-projects/coronavirus-government-response-tracker (2020). 10.1038/s41562-021-01079-8 33686204

[30] ACAPS. #COVID19 Government Measures Dataset. https://www.acaps.org/covid19-government-measures-dataset (2020).

[31] Zheng, Q. *et al.* HIT-COVID, a global database tracking public health interventions to COVID-19. *Sci. Data*10.1038/s41597-020-00610-2 (2020). 10.1038/s41597-020-00610-2 PMC7453020 32855428

[32] European Centre for Disease Prevention and Control. *Technical Report. Considerations Relating to Social Distancing Measures in Response to COVID-19 – Second Update* (ECDC, Stockholm, 2020).

[33] World Health Assembly. *International Health Regulations (2005)*. (World Health Organization, 2016).

[34] Kinlaw, K. & Levine, R. J. *Ethical guidelines in Pandemic Influenza—Recommendations of the Ethics Subcommittee of the Advisory Committee to the Director, Centers for Disease Control and Prevention*. (Centers for Disease Control and Prevention, 2007). 10.1097/DMP.0b013e3181ac194f 19675459

